# Endurance Exercise Prevented Diabetic Cardiomyopathy through the Inhibition of Fibrosis and Hypertrophy in Rats

**DOI:** 10.31083/j.rcm2505173

**Published:** 2024-05-16

**Authors:** Sadegh Shabab, Maryam Mahmoudabady, Zahra Gholamnezhad, Saeed Niazmand, Mahtab Fouladi, Zahra Mousavi Emadi

**Affiliations:** ^1^Department of Physiology, Faculty of Medicine, Mashhad University of Medical Sciences, 91779 48564 Mashhad, Iran; ^2^Applied Biomedical Research Center, Mashhad University of Medical Sciences, 91779 48564 Mashhad, Iran; ^3^Department of Pediatrics, Mashhad University of Medical Sciences, 91779 48564 Mashhad, Iran

**Keywords:** diabetes, cardiomyopathy, exercise, HIIT, MICT, hypertrophy, fibrosis

## Abstract

**Background:**

Exercise training could be essential in preventing pathological cardiac remodeling in diabetes. Therefore, the effects of moderate-intensity continuous training (MICT) and high-intensity interval training (HIIT) singly or plus metformin on diabetes-induced cardiomyopathy were investigated in this study.

**Methods:**

Forty-nine Wistar rats (male) were recruited. Seven groups of animals were treated for six weeks as control, diabetes, MICT (15 m/min, 40 min/day), HIIT (20 m/min, 40 min/day), metformin (300 mg/kg), HIIT+metformin (Met-HIIT), and MICT+metformin (Met-MICT). The metformin was orally administered with an intragastrical needle, and the exercised rats were trained (5 days/week) with a motorized treadmill. Metabolic parameters, echocardiographic indices, histopathology evaluation, and assessment of gene expression connected with cardiac fibrosis, hypertrophy, mitochondrial performance, and intracellular calcium homeostasis were investigated.

**Results:**

Our results demonstrated that all the interventions prevented weight loss and enhanced heart weight/body weight ratio and fasting plasma glucose in diabetic rats. Both types of exercise and their metformin combinations improved diabetic animals’ echocardiography indices by enhancing heart rate, fractional shortening (FS), ejection fraction (EF) and reducing end-systolic and end-diastolic diameter of left ventricular (LVESD and LVEDD). Gene expression of *atrial natriuretic peptide* (*ANP*), *brain natriuretic peptide* (*BNP*), *transforming growth factor* (*TGF*)*-β*, and *collagen* increased in the diabetes group. In contrast, the gene expression of *peroxisome proliferator-activated receptor gamma coactivator 1 alpha* (*PGC-1α*), *AMP-activated protein kinase* (*AMPK*), *ryanodine receptors* (*RyR*), and *${\mathrm{Ca}}^{2+}$ ATPase pump of the sarcoplasmic reticulum* (*SERCA*) was reduced in diabetic animals. Exercise training alone or in combination with metformin reversed these changes. Moreover, diabetes-induced cardiac fibrosis was ameliorated in treated groups. All indicators of diabetic cardiomyopathy were improved more in the Met-HIIT group than in other groups.

**Conclusions:**

Exercise training, notably with metformin combination, alleviated diabetes-induced cardiac complications. The beneficial effects of exercise could be related to improving pathological cardiac remodeling and enhancing cardiac function.

## 1. Introduction

One of the most fatal complications of diabetes is diabetic cardiomyopathy (DCM) [1]. DCM is defined as cardiac muscle dysfunction caused by diabetes, independent of hypertension and atherosclerosis [2]. It is estimated that 40 to 50 percent of diabetic patients suffer from heart disorders [3]. Although the exact induced mechanisms of DCM are unknown, the inflammation process, apoptosis, hypertrophy, and fibrosis contribute to the development of cardiac remodeling in diabetes [2]. Oxidative stress is caused by chronic hyperglycemia (the main complication of diabetes) and can lead to ventricular contractile dysfunction [4]. The elevation of reactive oxygen species (ROS) might activate the renin-angiotensin-aldosterone-system and signaling pathways like transforming growth factor beta (TGF)-β, which leads to hypertrophy and fibrosis in the diabetic heart [3, 5]. Cardiac fibrosis might reduce myocardial adjustment, resulting in failure of diastolic and systolic functions and, eventually, in cardiac contraction and pumping activity [6]. TGF-β1/Smad signaling has been shown to be an enhancer of collagen synthesis and interstitial fibrosis progression [6]. In addition, pathological hypertrophy of the heart following cardiac fibrosis is associated with the activation of fetal genes like *brain natriuretic peptide* (*BNP*) and *atrial natriuretic peptide* (*ANP*) and that enhances lengths or widths of cardiomyocytes and contributes to the development of DCM [3]. Mitochondrial dysfunction in various organs could induce cardiomyocyte energy insufficiency, reducing mitochondrial respiratory oxygen consumption [7]. *Peroxisome proliferator-activated receptor gamma coactivator 1 alpha* (*PGC-1α*) is pivotal in mitochondrial performance. The coactivator action of *PGC-1α* improves biogenesis, respiration, and transcriptional activation of mitochondria and reduces ROS generation and inflammatory processes in the vascular smooth muscle endothelial cells. *PGC-1α* levels depend on glycemic control and insulin sensitivity in skeletal muscle and myocardium [8]. *AMP-activated protein kinase* (*AMPK*) preserves ATP by retrieving the ratio of NAD+/NADH and excluding catabolic pathways, which will be turned on by reducing intracellular ATP levels [9]. Along the progression of DCM, the expression of some genes like the *${\mathrm{Ca}}^{2+}$ ATPase pump of the sarcoplasmic reticulum* (*SERCA*) and *ryanodine receptors* (*RyR*), which recreate an influential contribution in the normal operation of the heart muscle, might be disrupted [10].

Physical activity as a thrifty and non-pharmacological co-treatment is recommended as part of the rehabilitation for diabetic patients with cardiac disorders. Exercise can improve cardiac function by decreasing cardiac risk factors and myocardial damage in diabetes [11]. Furthermore, exercise could enhance mitochondrial biogenesis, ATP production, and cardiomyocyte contractility [12]. Physical activity has been shown to promote angiogenesis and vascular performance by attenuating oxidative stress and inflammatory processes [13]. The impact of exercise on diabetes-induced cardiovascular disorder might be related to the modalities, duration, and intensity level of training programs. The evidence showed that low and moderate-intensity exercise had been indicated to improve the metabolism of glucose and cellular apoptosis in the heart, which results in enhanced cardiac function [11].

Moreover, a cardiotonic and protective role for high-intensity exercise training has been demonstrated [11]. Both moderate-intensity continuous training (MICT) and high-intensity interval training (HIIT) modalities of exercise have been shown to decrease the glycosylated form of hemoglobin and fasting blood glucose (FBS) in patients with diabetes [13, 14]. However, HIIT training showed better outcomes in reducing FBS and ameliorating hyposensitivity to insulin in adults with diabetes [15, 16]. Furthermore, HIIT attenuated glucose and fatty acid metabolism, the respiratory capacity of mitochondria, and ventricular mechano-energetic coupling in cardiac tissue. At the same time, the MICT exercise could not improve this parameter [17, 18]. Exercise training in diabetes improves myocardial fibrosis by decreasing myocardial collagens and restoring fibrosis-related gene expression of matrix metalloproteinases [6]. Significant protective effects of exercise training in DCM have been revealed by improving *PGC-1α* and Akt signaling pathways and mitochondrial performance in cardiomyocytes in diabetes [19]. Activation of *AMPK* and improvement of glucose metabolism following exercise training are mentioned in animal and clinical trial studies [9]. Regulation of *RyR* and *SERCA*, as the main contributors in the removal of intracellular calcium, improved with exercise training [10]. Due to its hypoglycemic effect, metformin is one of the most commonly used oral first-line drugs in managing diabetes-induced hyperglycemia. It has also been demonstrated to have cardioprotective effects by diminishing the heart complications of diabetes. For these reasons, it was considered a positive control and was used simultaneously with exercise training [3, 20]. Altogether, less is known about the effects of either of these two exercise modalities on diabetes-induced cardiac fibrosis and hypertrophy. We hypothesized that the HIIT and MICT exercise may prevent pathological cardiac remodeling in diabetic rat models induced by streptozotocin (STZ). Therefore, the expression of *ANP* and *BNP* genes (as markers of cardiac hypertrophy), *TGF-β*and *collagen* genes (as markers of cardiac fibrosis), *RyR* and *SERCA* genes (as an indicator of intracellular calcium homeostasis), *PGC-1α* and *AMPK *genes (as an indicator of mitochondrial function) and echocardiography parameters were assessed to clarify the preventive effects of exercise on DCM.

## 2. Materials and Methods

### 2.1 Animals and Induction of Diabetes 

Male ten-week-old Wistar rats (weight of 250 ± 20 gr) were retrieved from the laboratory animal house of the faculty of medicine of Mashhad, Iran. They were kept in standard plastic rodent cages and had free access to water and a standard rodent diet. They were housed at the standard, light, and dark conditions (12 hr light/12 hr dark cycle), with 20–24 °C temperature and 40–60% humidity. One STZ dose (60 mg/kg, i.p.) was used for the induction of a diabetes model. The animals were considered diabetic if the FBS levels were detected over 250 mg/dL with a glucometer 72 hours after the STZ injection [3, 20].

### 2.2 Animals Therapeutic Protocols 

Rats (n = 49) were randomly split up into seven groups (n = 7) (Table 1). Animals recruited to a single intact control group (control; injected sterile saline, 1 mL/kg, i.p.) and six diabetic animal groups as follows: diabetic control group (Diabetes), diabetes treated with metformin (300 mg/kg) (Metformin), diabetes trained with HIIT exercise (HIIT), diabetes trained with MICT exercise (MICT), diabetes treated with metformin and trained with HIIT exercise (Met-HIIT), diabetes treated with metformin and trained with MICT exercise (Met-MICT). Treatment protocols were started after confirmation of diabetes induction (FBS >250 mg/dL) and continued for six weeks. The training exercise protocol was almost identical to our previous study [21]. A motorized treadmill with a zero-inclination angle was used for animal training. The rats’ exercise sessions were 40 minutes daily (5 days/week) for six consecutive weeks. On the first five days of training (familiarization period), rats were accustomed to the treadmill running at 12 to 15 m/min for 15 minutes daily. The protocol HIIT with a maximum speed of 20 m/min was determined by three 10-minute training periods alternately with 2-minute rest intervals between these training periods. The protocol MICT was defined at a continuous speed of 15 meters per minute. The first and last 3 minutes of the training duration were dedicated to warming and cooling the rats, respectively, at a speed of 12 m/min [13, 16, 21, 22, 23]. At the end of the experiments, after performing echocardiography, deep anesthesia with xylazine and ketamine (8 mg/kg and 60 mg/kg, respectively, i.p.) was applied to the rats for painless sacrificing. After opening the chest, blood samples were obtained from the heart for biochemical assessments. Subsequently, the harvested heart was washed in cold saline and weighed, and then the left ventricle tissue was divided into two parts. The apex was kept in RNA later for gene expression assay, and the residual part was used for histological evaluation [3, 20].

**Table 1.** **Experimental protocol in rats of different groups**.

Groups (number of rats = 7)	Treatment protocols
Group I (Control)	—–
Group II (Diabetes)	—–
Group III (Metformin)	Metformin (300 mg/kg)
Group IV (HIIT)	HIIT (High-intensity interval training)
Group V (MICT)	MICT (Moderate-intensity continuous training)
Group VI (Met-HIIT)	HIIT exercise + Metformin (300 mg/kg)
Group VII (Met-MICT)	MICT exercise + Metformin (300 mg/kg)

Diabetes + metformin (Metformin), diabetes + HIIT exercise training (HIIT), diabetes + MICT exercise training (MICT), diabetes + metformin + HIIT exercise training (Met-HIIT), diabetes + metformin + MICT exercise training (Met-MICT). HIIT, high-intensity interval training; MICT, moderate-intensity continuous training.

### 2.3 Left Ventricular Function

Echocardiography was performed to survey heart function using a neonatal echocardiographic device (12-MHz linear probe). At first, the rats underwent light anesthesia with the intraperitoneal injection of low dose xylazine + ketamine/(2 mg/kg and 10 mg/kg respectively, i.p.). The left ventricular end-systolic diameter (LVESD), left ventricular end-diastolic diameter (LVEDD), and heart rate (HR) indices of the left ventricle in the animals were measured. Also, according to the previous study, fractional shortening (FS) and ejection fraction (EF) indices were obtained through the standard formulas [24].

### 2.4 Histopathological Studies

After preparing and processing cardiac tissue, the Masson trichrome staining was applied to identify fibrosis. A double-blinded researcher assessed the images with a light microscope (Nikon Eclipse E200, Tokyo, Japan). Two examiners, blinded to the animal groups, analyzed ten randomly selected fields on each slide for seven rats per group; the blue color stain identified collagen fibers. Cardiac fibrosis was determined according to data from ten randomly selected high-power fields (400X) for each tissue section. The image J software (Version: 1.53f51, NIH, Bethesda, MD, USA) was chosen to evaluate collagen percentage [21, 24].

### 2.5 Quantitative Real-Time Polymerase Chain Reaction

To perform RNA extraction, the cardiac tissue was homogenized with Trizol (Yekta Tajhiz Azma Co, Tehran, Iran). The quality and purity of the harvested RNA were detected using a nanodrop 2000 (Thermo Scientific, Waltham, MA, USA). The cDNA was synthesized with the easy cDNA kit (Parstous, Mashhad, Iran) using a BioRad C1000 thermal cycler (Bio-Rad, Hercules, CA, USA). The quantitative real-time polymerase chain reaction (qRT-PCR) was conducted using the Light Cycler System (Roche Diagnostics, Mannheim, Germany) and Ampliqon Real Q Plus 2x Master Mix Green (Ampliqon, Odense, Denmark) to investigate the gene expressions. β*-actin* was used as a housekeeping gene for internal control. The gene sequences were obtained and approved with the NCBI Gene database. Based on previous studies, the changes in gene expression were calculated using the Fold Change formulation. The mRNA sequences are presented in Table 2 [20, 24].

**Table 2.** **Target mRNAs sequences**.

Gene	Primer sequence $5^{\prime }$${\mathrm{–3}}^{\prime }$
*Beta-Actin*	F-CCCGCGAGTACAACCTTCT
	R-CCATCACACCCTGGTGCCTA
*Collagen*	F-TGCCGTGACCTCAAGATGTG
	R-TCTGACCTGTCTCCATGTTGC
*TGF-β*	F-GCTACCATGCCAACTTCTGTCT
	R-CCTACCACCCCAGCCTCTG
*ANP*	F-CTCCATCACCAAGGGCTTCTTC
	R-ATCTGTGTTGGACACCGCACTG
*BNP*	F-CCAGAACAATCCACGATGCAG
	R-TTGTAGGGCCTTGGTCCTTTG
*PGC1-α*	F-CGCAGGTCGAATGAAACTGAC
	R-GTGGAAGCAGGGTCAAAATCG
*AMPK*	F-CCCTTGAAGCGAGCAACTATC
	R-AGCATCATAGGAGGGGTCTTC
*SERCA*	F-ACGAGACGCTCAAGTTTGTGG
	R-GCTAACAACGCACATGCAC
*RyR*	F-CGAATCAGTGAACGCCAAGG
	R-CCTGCTCGGTCAGCTCTAAG

*TGF-β*, *transforming growth factor-β*; *ANP*, *atrial natriuretic peptide*; *BNP*, *brain natriuretic peptide*; *PGC-1α*, *peroxisome proliferator-activated receptor gamma coactivator 1 alpha*; *AMPK*, *AMP-activated protein kinase*; *SERCA*, *${\mathrm{Ca}}^{2+}$ ATPase pump of the sarcoplasmic reticulum*; *RyR*, *ryanodine receptors*.

### 2.6 Statistical Analysis

Acquired data are represented as mean ± standard error of the mean (SEM). The SPSS program version 20.0 (IBM SPSS Inc., Armonk, NY, USA) was used for statistical analysis. One-way analysis of variance (ANOVA) was used to compare groups, followed by Tukey’s *post-hoc* test. *p* < 0.05 was determined as a statistically significant level.

## 3. Results

### 3.1 Markers of Metabolism Abnormalities

The data showed that the body weight (BW) was significantly decreased in diabetic animals. In contrast, the ratio of heart weight to body weight (HW/BW) and FBS increased dramatically in the diabetic group compared to the control rats (*p* < 0.001). The BW in Met-MICT and metformin groups significantly increased compared to the diabetes group (*p* < 0.01, *p* < 0.05, respectively). The HW/BW significantly reduced in all treatment groups compared to diabetes rats (*p* < 0.001). This ratio in the Met-MICT group did not show a significant difference compared to the control group. The FBS level in all treatment groups was considerably lower than the diabetes group (*p* < 0.001). There was a significant reduction in FBS levels in the Met-HIIT, Met-MICT, and metformin groups than the MICT group (*p* < 0.001). There was no significant difference in FBS levels between the Met-HIIT and the control groups (Table 3).

**Table 3.** **Comparison of the metabolic parameters in experimental groups**.

Groups	BW (g)	HW/BW (mg/g)	FBS (mg/dL)
Control	320 ± 6.18	3.06 ± 0.22	104 ± 9.93
Diabetes	230 ± ${\mathrm{7.48}}^{\text{c}}$	5.24 ± ${\mathrm{0.19}}^{\text{c}}$	512 ± ${\mathrm{21.76}}^{\text{c}}$
HIIT	255 ± ${\mathrm{13.54}}^{\text{c}}$	3.73 ± ${\mathrm{0.09}}^{\text{bf}}$	236 ± ${\mathrm{17.00}}^{\text{cf}}$
MICT	245 ± ${\mathrm{14.30}}^{\text{c}}$	4.07 ± ${\mathrm{0.20}}^{\text{cf}}$	352 ± ${\mathrm{23.07}}^{\text{cfi}}$
Met-HIIT	234 ± ${\mathrm{7.22}}^{\text{c}}$	4.07 ± ${\mathrm{0.19}}^{\text{cf}}$	166 ± ${\mathrm{16.18}}^{\text{fl}}$
Met-MICT	270 ± ${\mathrm{6.40}}^{\text{ce}}$	3.51 ± ${\mathrm{0.08}}^{\text{fj}}$	197 ± ${\mathrm{20.88}}^{\text{bfl}}$
Metformin	259 ± ${\mathrm{8.88}}^{\text{cd}}$	3.63 ± ${\mathrm{0.05}}^{\text{af}}$	190 ± ${\mathrm{10.32}}^{\text{bfl}}$

Data are represented as mean ± SEM (n = 7). ^a^*p* < 0.05, ^b^*p* < 0.01, ^c^*p* < 0.001 vs control group; ^d^*p* < 0.05, ^e^*p* < 0.01, ^f^*p* < 0.001 vs diabetic group; ^i^*p* < 0.001 vs HIIT group; ^j^*p* < 0.05, ^1^*p* < 0.001 vs MICT group. BW, body weight; HW, heart weight; FBS, fasting blood sugar; HIIT, high-intensity interval training; MICT, moderate-intensity continuous training; Met-HIIT, HIIT+metformin; Met-MICT, MICT+metformin; SEM, standard error of the mean.

### 3.2 Left Ventricular Dysfunction Indices

HR in the diabetes group (351 ± 7 bpm) was lower than the control group (474 ± 11 bpm) (*p* < 0.001). There was a significant increase in heart rate in all treatment groups compared to the diabetes group (*p* < 0.01 to *p* < 0.001). Data showed an increase in LVEDD and LVESD in the diabetes group (6.33 ± 0.35 mm and 6.07 ± 0.24 mm, respectively) in comparison to the control group (5.16 ± 0.23 mm and 4.05 ± 0.14 mm, respectively) (*p* < 0.01 and *p* < 0.001, respectively). Functional indicators of EF and FS were reduced in diabetic animals (52.2 ± 2.00% and 33.5 ± 1.29%, respectively) compared to control ones (81.2 ± 1.70% and 46.6 ± 1.77%, respectively) (*p* < 0.001). Both combined treatment groups and the metformin group showed improvements in indicators of LVEDD and LVESD (*p* < 0.05 to *p* < 0.001) compared to the diabetes group. Also, the HIIT exercise improved the LVESD index (5.51 ± 0.20 mm) in comparison with the diabetes group (*p* < 0.05). EF percentage in all training groups and the metformin group exhibited higher values compared to the diabetes group (*p* < 0.01 to *p* < 0.001). The improvement of FS percentage was observed in groups of HIIT, Met-HIIT, and metformin compared to the diabetic group (*p* < 0.05 to *p* < 0.001) (Table 4).

**Table 4.** **The echocardiography indices in experimental groups**.

Group	HR (bpm)	LVEDD (mm)	LVESD (mm)	EF (%)	FS (%)
Control	474 ± 11	5.16 ± 0.23	4.05 ± 0.14	81.2 ± 1.70	46.6 ± 1.77
Diabetes	351 ± $7^{\text{c}}$	6.33 ± ${\mathrm{0.35}}^{\text{b}}$	6.07 ± ${\mathrm{0.24}}^{\text{c}}$	52.2 ± ${\mathrm{2.00}}^{\text{c}}$	33.5 ± ${\mathrm{1.29}}^{\text{c}}$
HIIT	417 ± ${\mathrm{13}}^{\text{cf}}$	5.96 ± ${\mathrm{0.22}}^{\text{a}}$	5.51 ± ${\mathrm{0.20}}^{\text{cd}}$	85.3 ± ${\mathrm{3.93}}^{\text{f}}$	57.9 ± ${\mathrm{2.08}}^{\text{cf}}$
MICT	393 ± $8^{\text{ce}}$	5.99 ± ${\mathrm{0.20}}^{\text{a}}$	5.76 ± ${\mathrm{0.19}}^{\text{c}}$	65.8 ± ${\mathrm{3.15}}^{\text{bei}}$	39.9 ± ${\mathrm{1.22}}^{\text{i}}$
Met-HIIT	404 ± ${\mathrm{11}}^{\text{cf}}$	5.28 ± ${\mathrm{0.17}}^{\text{ej}}$	4.89 ± ${\mathrm{0.19}}^{\text{bfgl}}$	73.2 ± ${\mathrm{2.52}}^{\text{fg}}$	44.1 ± ${\mathrm{2.45}}^{\text{ei}}$
Met-MICT	403 ± ${\mathrm{11}}^{\text{cf}}$	5.35 ± ${\mathrm{0.25}}^{\text{e}}$	5.12 ± ${\mathrm{0.13}}^{\text{cfj}}$	68.6 ± ${\mathrm{2.24}}^{\text{afi}}$	41.1 ± ${\mathrm{1.43}}^{\text{i}}$
Metformin	394 ± $6^{\text{ce}}$	5.47 ± ${\mathrm{0.27}}^{\text{d}}$	5.27 ± ${\mathrm{0.16}}^{\text{ce}}$	71.8 ± ${\mathrm{1.06}}^{\text{fh}}$	41.5 ± ${\mathrm{2.02}}^{\text{di}}$

Data are represented as mean ± SEM (n = 7). ^a^*p* < 0.05, ^b^*p* < 0.01, ^c^*p* < 0.001 vs control group; ^d^*p* < 0.05, ^e^*p* < 0.01, ^f^*p* < 0.001 vs diabetic group; ^g^*p* < 0.05, ^h^*p* < 0.01, ^i^*p* < 0.001 vs HIIT group; ^j^*p* < 0.05, ^l^*p* < 0.001 vs MICT group. HR, heart rate; LVEDD, left ventricular end-diastolic diameter; LVESD, left ventricular end-systolic diameter; EF, ejection fraction; FS, fractional shortening; SEM, standard error of the mean.

### 3.3 Cardiac Pathological Changes

**Fig. 1.**
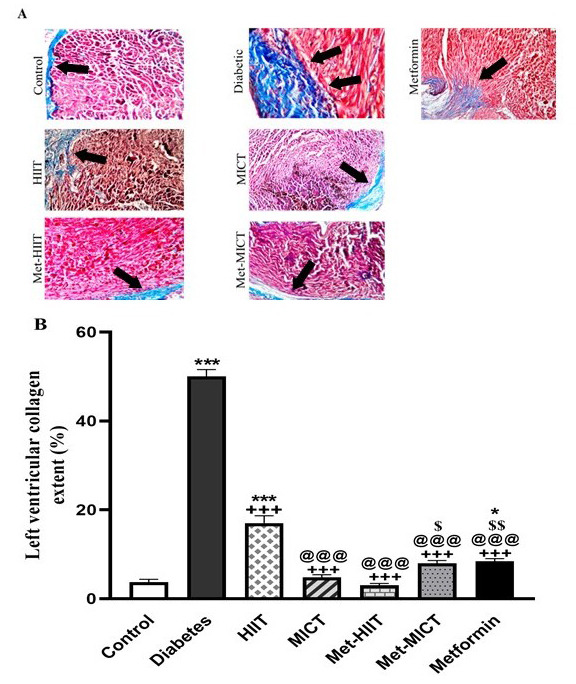
** Comparison of the cardiac pathological changes in different groups of study**. (A) Representative images of cardiac tissue sections stained with the Masson trichrome. The arrow indicates myocardial fibrosis stained in blue. (B) Quantitative analysis of fibrosis area. The data are expressed as mean ± SEM (n = 7). *p < 0.05, ***p < 0.001 vs control group; +++*p* < 0.001 vs diabetes group; @@@*p* < 0.001 vs HIIT group; $*p* < 0.05, $$*p* < 0.01 vs Met-HIIT; HIIT, high-intensity interval training; MICT, moderate-intensity continuous training; Met-HIIT, HIIT+metformin; Met-MICT, MICT+metformin; SEM, standard error of the mean.

The ventricular muscle had typical structures in the control animals. The heart tissue of the diabetic rats had prominent fibrosis without regular patterns that it demonstrated with interstitial collagen structure. The data showed that in the diabetic group, there was a significant collagen deposition compared to control rats in the left ventricular tissue (*p* < 0.001). The intervention groups had significantly less fibrotic tissue compared to the diabetic hearts with no intervention, and it was the most evident in the Met-HIIT group (*p* < 0.001) (Fig. 1).

### 3.4 Cardiac Hypertrophy Marker Genes

**Fig. 2.**
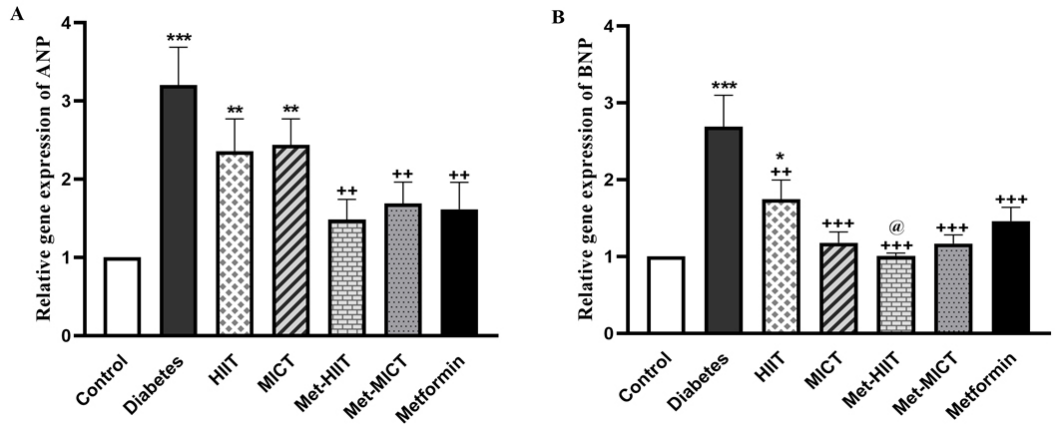
** Comparison of the *AN*P (A) and *BNP* (B) gene expression in different groups of study**. The data are shown as mean ± SEM (n = 7). *p < 0.05, **p < 0.01, ***p < 0.001 vs control group; ++*p* < 0.01, +++*p* < 0.001 vs diabetes group; @*p* < 0.05 vs HIIT group. HIIT, high-intensity interval training; MICT, moderate-intensity continuous training; Met-HIIT, HIIT+metformin; Met-MICT, MICT+metformin; *ANP*, *atrial natriuretic peptide*; *BNP*, *brain natriuretic peptide*; SEM, standard error of the mean.

The expression of *ANP* and *BNP* was upregulated in the diabetes group compared to the control group (*p* < 0.001). Although exercise training reduced the expression of *ANP* in diabetic animals, it was significantly higher than the control group (*p* < 0.01). Treating animals with metformin and its combination with exercise significantly diminished *ANP* expression compared to the diabetes group (*p* < 0.01). All the intervention groups had a considerably reduced BNP expression compared to the diabetic group (*p* < 0.01 to *p* < 0.001). There was no significant difference in *BNP* expression between the intervention and control groups except for the HIIT group (*p* < 0.05). Moreover, the best response regarding decreased *BNP* expression to the control groups’ level was related to the Met-HIIT group (Fig. 2A,B).

### 3.5 Cardiac Fibrosis Marker Genes

**Fig. 3.**
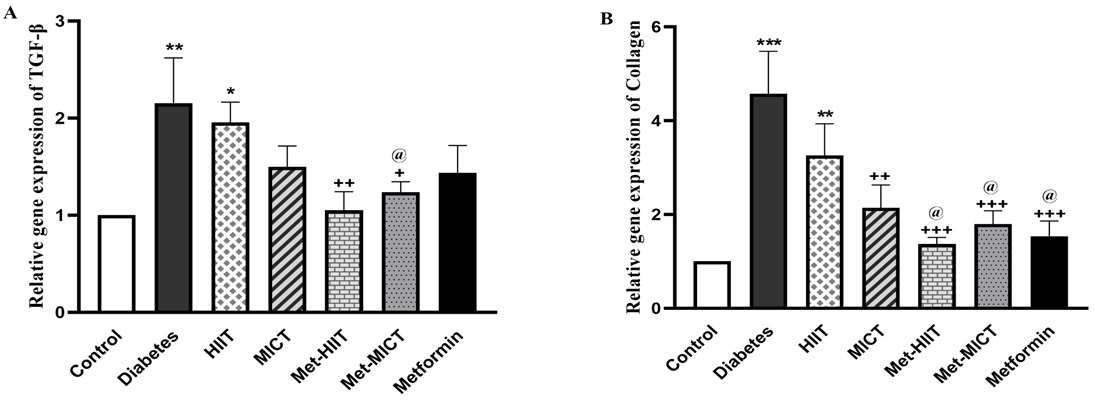
**Comparison of the gene expression of *TGF-β* (A) and *collagen* (B) in different groups of study**. The data are expressed as mean ± SEM (n = 7). *p < 0.05, **p < 0.01, ***p < 0.001 vs control group; +*p* < 0.05, ++*p* < 0.01, +++*p* < 0.001 vs diabetes group; @*p* < 0.05 vs HIIT group. HIIT, high-intensity interval training; MICT, moderate-intensity continuous training; Met-HIIT, HIIT+metformin; Met-MICT, MICT+metformin; *TGF-β*, *transforming growth factor-β*; SEM, standard error of the mean.

The relative *TGF-β* and *collagen* gene expression in the heart of the diabetic group increased significantly in comparison to the control group (*p* < 0.01–*p* < 0.001). The level of *TGF-β* expression in the combination groups showed a significant decline compared to the diabetes group (*p* < 0.05–*p* < 0.01). The best response regarding the reduction of *TGF-β* gene expression was observed in the Met-HIIT group (*p* < 0.01). The collagen gene expression in all treatment groups significantly reduced compared to the diabetes group, except in the HIIT group (*p* < 0.01–*p* < 0.001). The expression of both of these fibrotic genes in the Met-HIIT group was closer to the control group (Fig. 3A,B).

### 3.6 The Associated Genes of Mitochondrial Function

**Fig. 4.**
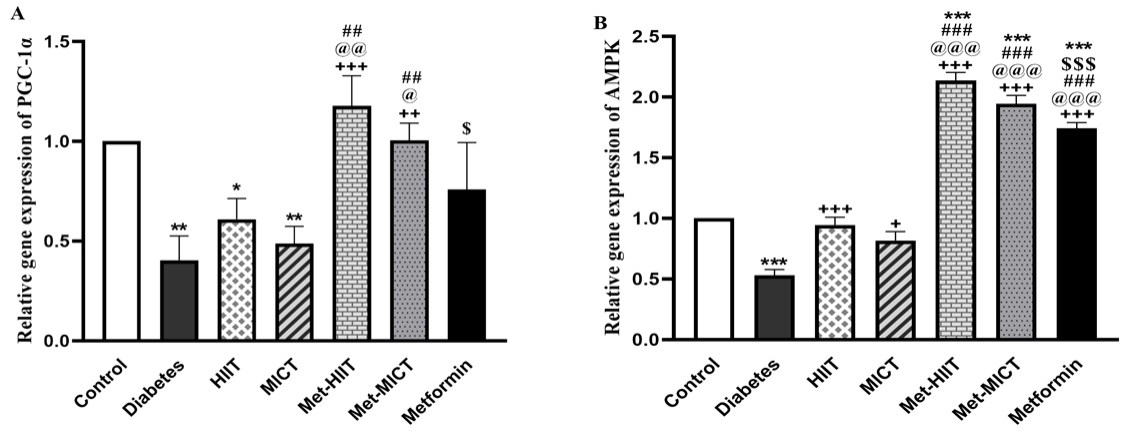
**Comparison of the gene expression of *PGC-1α* (A) and *AMPK *(B) in different groups of study**. The data are expressed as mean ± SEM (n = 7). *p < 0.05, **p < 0.01, ***p < 0.001 vs control group; +*p* < 0.05, ++*p* < 0.01, +++*p* < 0.001 vs diabetes group; @*p* < 0.05, @@*p* < 0.01, @@@*p* < 0.001 vs HIIT group; ##*p* < 0.01, ###*p* < 0.001 vs MICT; $*p* < 0.05 vs Met-HIIT group, $$$*p* < 0.001 vs Met-HIIT group. HIIT, high-intensity interval training; MICT, moderate-intensity continuous training; Met-HIIT, HIIT+metformin; Met-MICT, MICT+metformin; *PGC-1α*, *peroxisome proliferator-activated receptor gamma coactivator 1 alpha*; *AMPK*, *AMP-activated protein kinase*; SEM, standard error of the mean.

The expression of *PGC-1α* declined in the diabetes group compared to the control group (*p* < 0.01). Although all of the intervention groups showed an increase in the expression of the *PGC-1α* gene compared to the diabetes group, this difference was only significant in combined groups (*p* <0.01–*p* < 0.001). This increase was more pronounced in the Met-HIIT group. Furthermore, the expression of *AMPK *was decreased in the diabetes group compared to the control group (*p* < 0.001). When compared to the diabetes group, the expression of the *AMPK *gene was considerably increased in all the treatment groups (*p* < 0.05 and *p* < 0.001) with the superiority of the Met-HIIT group (Fig. 4A,B).

### 3.7 The Associated Genes of Calcium Metabolism

**Fig. 5.**
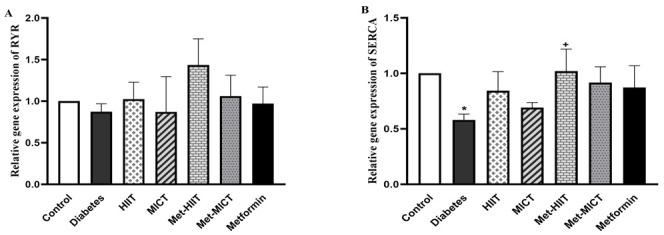
**Comparison of the gene expression of *RyR* (A) and *SERCA* (B) in different groups of study**. The data are expressed as mean ± SEM (n = 7). *p < 0.05 vs control group; +*p* < 0.05 vs diabetes group. HIIT, high-intensity interval training; MICT, moderate-intensity continuous training; Met-HIIT, HIIT+metformin; Met-MICT, MICT+metformin; *SERCA*, *${\mathrm{Ca}}^{2+}$ ATPase pump of the sarcoplasmic reticulum*; *RyR*, *ryanodine receptors*; SEM, standard error of the mean.

The gene expression of *RyR *was not significantly changed in the diabetes vs control group. Also, the upregulation of the *RyR* gene expression in all the treatment groups was insignificant compared to the diabetes group. However, it is worth noting that the Met-HIIT group had the most increase in the *RyR* gene expression compared to the diabetic ones. The expression of *SERCA* was reduced in the diabetes group compared to the control group (*p* < 0.05). Although all of the intervention groups showed an increase in the expression of the *SERCA* gene compared to the diabetes group, this difference was only statistically significant in the Met-HIIT group (*p* < 0.05) (Fig. 5A,B).

### 3.8 Correlation between the Expression of Fibrotic and Hypertrophic Genes with Echocardiographic Parameters

**Fig. 6.**
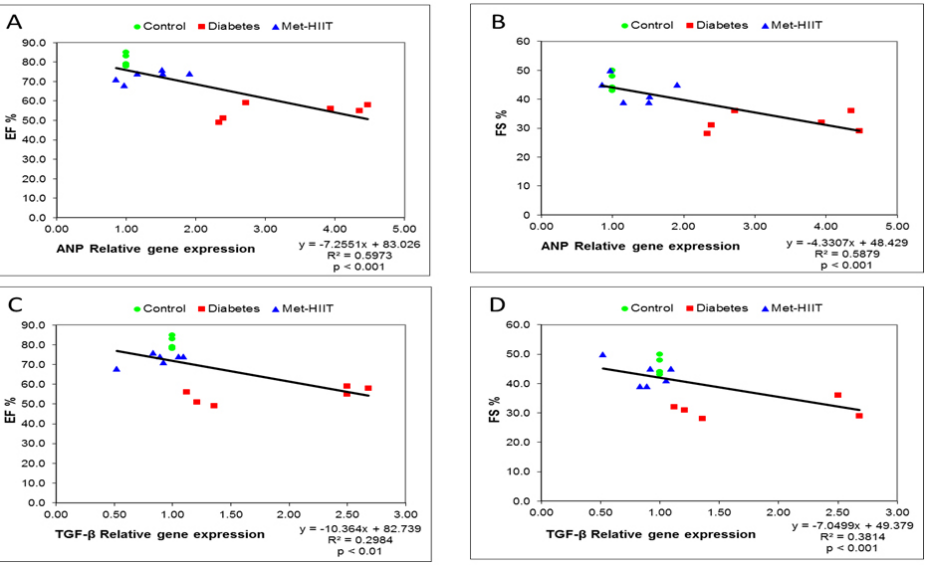
**Scatterplots of *ANP *(A,B) and *TGF-β* (C,D) (x-axis) expressing genes in the association with the echocardiographic indices (y-axis)**. TGF-β, *transforming growth factor-β*; *ANP*, *atrial natriuretic peptide*; EF, ejection fraction; FS, fractional shortening; HIIT, high-intensity interval training; Met-HIIT, HIIT+metformin.

Fig. 6 shows a significant relationship between the expression of the *ANP* gene as a hypertrophic marker and the *TGF-β* gene as a fibrosis marker of the heart with the echocardiographic indices of EF and FS.

## 4. Discussion

Although cardiomyopathy caused by diabetes does not have a precise and proven mechanism, pathological cardiac remodeling caused by inflammatory processes, fibrosis, and hypertrophy could be a principal cause of the development of this disorder [2]. The pathological cardiac remodeling due to fibrosis is induced by extracellular matrix accumulation and upregulation of *TGF-β* as the main profibrotic factor. *TGF-β1* could be activated by oxidative stress, following binding to its receptors and triggering collagen production through the smad signaling pathway [4]. Due to chronic hyperglycemia in diabetic patients, up-regulation of the fetal proteins related to hypertrophy, such as *ANP* and *BNP* in the cardiomyocytes, increases dramatically [24]. Moreover, the disturbed mitochondrial performance is involved in the DCM pathophysiological mechanisms, which could be regulated with *PGC-1α* [19]. Other signaling pathway activations like the *AMPK* in response to energetic demand and calcium-based myocardial performance mediated by *RyR2* and *SERCA2* are among the implicated parameters in DCM [10, 25]. The exercise protocols might alleviate cardiovascular complications and mitigate DCM incidence by preventing pathological cardiac remodeling.

Nevertheless, we studied the impact of two exercise modalities in preventing cardiac complications following diabetes. Metformin, a pharmacological diabetic treatment with a cardioprotective effect, was considered a positive control [24]. We observed that exercise with or without metformin and improving hyperglycemia inhibited fibrosis and hypertrophy by modifying the expression of target genes contributing to this process. Echocardiographic and histological data also confirmed this alleviation. Likewise, the improvement in the expression of genes that contributed to mitochondrial function and homeostasis of intracellular calcium after exercise treatment was in favor of the positive effect of exercise in inhibiting pathological cardiac remodeling in the course of diabetes.

As in previous similar experimental and clinical investigations, FBS levels in diabetic rats were significantly reduced following exercise training, although this reduction was more pronounced in the Met-HIIT group [23, 26, 27, 28, 29]. However, another study indicated the beneficial effect of MICT in lowering blood sugar in diabetic rats [13]. Exercise training might restore hyperglycemia by enhancing the muscle blood flow and ameliorating mitochondrial function. Additionally, its mechanism may be related to enhancing insulin sensitivity, which might improve glucose uptake [22, 29, 30].

Cardiac fibrosis might disturb myocardial adjustment and cardiac contraction, which leads to cardiomyopathy development and heart failure [6]. Here, we revealed that the exercise alleviated the expression of *TGF-β* and *collagen* genes in the diabetic group; the best response belonged to the Met-HIIT-treated rats. Likewise, the enhancement in these fibrotic factors has been reported in different study models of diabetic hearts and cardiac infarction [4, 6, 13, 31]. The TGF-β1/Smad signaling pathway is related to collagen synthesis and interstitial fibrosis progression. Nonetheless, alleviated TGF-β and collagen gene expression in treated animals might reflect that exercise training may mitigate fibrosis in cardiac tissue by inhibiting the molecular mechanism such as renin-angiotensin-aldosterone-system that leads to the prevention of myocardial collagen deposition and consequently cardiomyopathy in diabetes [4, 6]. The modulation of *TGF-β* and *collagen* gene expression was compatible with changes in the histopathological pattern associated with fibrosis. According to our histopathological evaluation and consistent with previous evidence, it can be supposed that exercise training intervention in diabetic cases can act as an anti-fibrotic factor by reducing collagen accumulation [6, 27, 32].

Pathological hypertrophy following cardiac fibrosis is associated with the over-expression of fetal hypertrophic genes that enhance the lengths or widths of cardiomyocytes and develop DCM [3]. Improvement of cardiac hypertrophy by reversing *ANP* and *BNP* gene expression has been indicated after exercise conditioning in diabetic animals [33]. In this regard, we observed that in the Met-HIIT group, the expression of these genes decreased more and became closer to the control group. The changes in the echocardiographic data mirrored this. Lowered expression of the hypertrophy hallmark genes, in addition to restoring the echocardiographic indices of LVEDD and LVESD in exercise groups, indicate that exercise training might prevent DCM by improving cardiac hypertrophy in the course of diabetes.

Nevertheless, exercise conditioning has been demonstrated to fail to boost the cardiac hypertrophy of diabetic *db/db* mice in a study, but metformin administration reversed cardiac hypertrophy. Consequently, cardiac hypertrophy in the early stages of cardiomyopathy is more related to glucose metabolism abnormality [34]. It is worth noting that in this work, exercise training prevented weight loss and decreased HW/BW index in diabetic animals. It has been shown that exercise might improve glucose metabolism and prevent diabetic ketoacidosis and weight loss by increasing the skeletal muscle and liver response to insulin and reducing fat oxidation [16, 29]. The effectiveness of exercise training may improve cardiac performance by attenuating fibrosis and hypertrophy in the heart, which results in the prohibition of cardiac disturbance induced by diabetes. It also can be a reasonable justification for these changes, as mentioned by previous studies [6, 13, 16].

Mitochondrial dysfunction in myocardial cells is another complication caused by diabetes. The level of *PGC-1α* gene expression, as a pivotal element contributing to mitochondrial function, has been decreased in heart failure [8, 19]. The ability of exercise training as an ameliorative factor for this cellular function by upregulating the expression of the *PGC-1α* has been previously reported [8, 19, 35, 36]. We also observed over-expression of this gene in exercise-treated groups. Meanwhile, the expression was higher in the combined group of Met-HIIT. *PGC-1α*, as a primary modifier in the biogenesis of mitochondria, has a potential role in the energy metabolism of the myocardium. The researchers indicated that exercise training improves DCM through ameliorating cardiac performance associated with restoring mitochondrial biogenesis, accompanied by *PGC-1α* activation and Akt signaling [19]. *AMPK* is an essential cellular pathway turned on due to bio-energetic demand and physical activity [25]. Aerobic exercise might increase *AMPK* gene expression and phosphorylation in diabetic animals [33]. The expression level of the *AMPK* gene was considerably increased in exercise groups compared to diabetic animals, especially in the Met-HIIT group. The preventive role of exercise against DCM in diabetescould be increasing *AMPK* associated with downregulating forkhead box transcription factors 1 (FOXO1) as a downstream effector of *AMPK * [33]. These changes in gene expression related to mitochondrial function, which improves cellular energy supply, have been accompanied by increased EF and FS performance indicators. During the progression of diabetes, heart diastolic dysfunction in the absence of hypertension can inevitably confirm the disorder of intracellular calcium homeostasis [34]. Severe systolic dysfunction was also demonstrated in diabetic patients, which might be related to the alteration of gene expression involved in adjusting intracellular calcium homeostasis. The changeover of calcium-based activities such as RyR2 and SERCA2a sensitivity change in diabetesis somewhat responsible for the myocardial contraction disturbance [10].

Modulating *RyR* and *SERCA* mRNA expression and their protein levels following endurance exercise protocols, which were reduced in diabetic rats, may also improve systolic and diastolic dysfunction in diabetic cardiomyopathy [10, 37]. Our data did not show a significant change in the expression levels of the *RyR* gene in all of the treated groups; meanwhile, in the Met-HIIT treated animals, there was an increase in the expression level of the *SERCA* gene. This upregulation could be attributed to the impact of exercise conditioning in enhancing cardiac performance in terms of echocardiographic parameters EF and FS.

Moreover, we showed a significant correlation between the gene expression level of *ANP* as an index of hypertrophy in cardiac tissue and *TGF-β* as a fibrotic index within the echocardiographic indices of EF and FS, which indicated a close association between the expression level of the mentioned genes and cardiac function. This significant relationship emphasizes the positive effect of simultaneous administration of metformin and HIIT exercise treatment on improving the prevention of pathological cardiac remodeling.

It should be mentioned that our findings cannot suggest which type of exercise is more effective for diabetes management. According to the effectiveness of exercise type, some parameters, including EF and FS in the echocardiography and the expression of the *AMPK* gene, were improved more in the HIIT-exercised rats than the MICT one, at the same time, reduced the percentage of collagen content in the MICT-exercised animals than the HIIT-exercised group. Controversial findings were also mentioned in previous studies [29, 38].

Considering that the progression of fibrosis and hypertrophy are the most critical mechanisms in the development of cardiomyopathy and diastolic dysfunction in diabetic patients, it seems that recommending physical exercises in addition to common diabetes treatments can prevent or delay pathological remodeling of the heart.

### Limitations

As a limitation, the induction of diabetes in animals could not mimic all features of cardiomyopathy in diabetic patients. In addition, maximal oxygen consumption (VO2 max) was not determined in this study, and a performance test evaluated exercise intensity. Although in the present experiment, HIIT exercise resulted in a better outcome for ameliorating cardiomyopathy, however for diabetic patients, the exercise intensity and duration should be determined individually based on pathological condition.

Furthermore, some clinical echocardiographic indices could not be evaluated in the rodents. Since our results were obtained from experimental animal studies, which differ from those of humans in some aspects, more evidence is needed to apply such findings in clinics. Additionally, complementary molecular investigations such as western blot and immunohistochemistry could be considered in future studies for better elucidation of DCM signaling pathways.

## 5. Conclusions

**Fig. 7.**
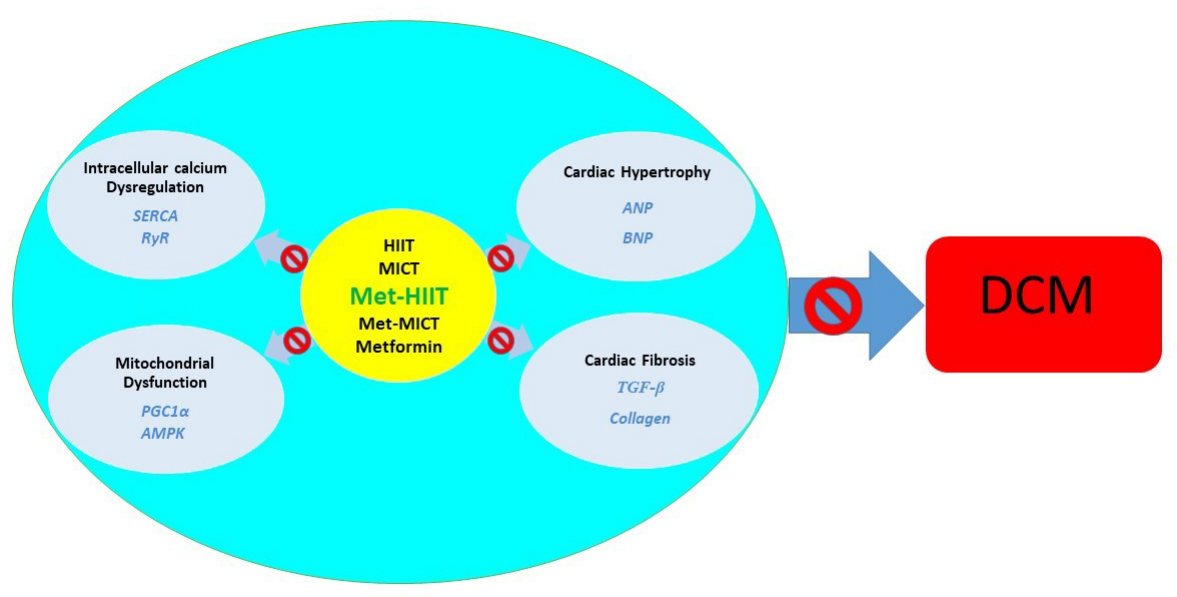
**The impact of exercise training on diabetic cardiomyopathy mechanisms**. *TGF-β*, *transforming growth factor-β*; *ANP*, *atrial natriuretic peptide*; *BNP*, *brain natriuretic peptide*; *PGC-1α*, *peroxisome proliferator-activated receptor gamma coactivator 1 alpha*; *AMPK*, *AMP-activated protein kinase*; *SERCA*, *${\mathrm{Ca}}^{2+}$ ATPase pump of the sarcoplasmic reticulum*; *RyR*, *ryanodine receptors*; HIIT, high-intensity interval training; MICT, moderate-intensity continuous training; Met-HIIT, HIIT+metformin; Met-MICT, MICT+metformin; DCM, diabetic cardiomyopathy.

The data showed that exercise training, especially in combination with metformin, in addition to improving hyperglycemia, prevents cardiomyopathy in diabetic rats by attenuating cardiac hypertrophy and fibrosis and maintaining mitochondrial function and intracellular calcium homeostasis (Fig. 7). These effects were observed more prominently in the Met-HIIT group, related to the type of exercise training protocols. Our findings increase our understanding of the benefits of physical activity on diabetes-induced cardiovascular disease and provide a practical target for DCM prevention. These results showed that exercise, especially with an anti-diabetic drug such as metformin, can be included in the rehabilitation therapy of diabetic patients with cardiovascular complications.

## Data Availability

Data will be made available on request, and the corresponding author can be contacted if needed.
